# Brain Structure and Function in Women with Comorbid Bipolar and Premenstrual Dysphoric Disorder

**DOI:** 10.3389/fpsyt.2017.00301

**Published:** 2018-01-10

**Authors:** Sabrina K. Syan, Luciano Minuzzi, Mara Smith, Dustin Costescu, Olivia R. Allega, Geoffrey B. C. Hall, Benicio N. Frey

**Affiliations:** ^1^MiNDS Neuroscience Graduate Program, McMaster University, Hamilton, ON, Canada; ^2^Women’s Health Concerns Clinic, St. Joseph’s Healthcare Hamilton, Hamilton, ON, Canada; ^3^Mood Disorders Program, St. Joseph’s Healthcare Hamilton, Hamilton, ON, Canada; ^4^Department of Psychiatry and Behavioural Neurosciences, McMaster University, Hamilton, ON, Canada; ^5^Department of Obstetrics and Gynecology, McMaster University, Hamilton, ON, Canada; ^6^Department of Psychology, Neuroscience and Behaviour, McMaster University, Hamilton, ON, Canada

**Keywords:** fMRI, bipolar disorder, premenstrual dysphoric disorder, MRI, menstrual

## Abstract

**Introduction:**

Hormonal fluctuations associated with female reproductive life events may precipitate or worsen affective episodes in women with bipolar disorder (BD). Previous studies have shown that women with BD report higher rates of premenstrual dysphoric disorder (PMDD) than controls. Further, bipolar women who report premenstrual worsening of mood display a worse course of their bipolar illness. Despite this, the neural correlates of comorbid BD and PMDD have not been investigated.

**Methodology:**

Eighty-five [CTRL, *n* = 25; PMDD, *n* = 20; BD, *n* = 21; BD with comorbid PMDD (BDPMDD), *n* = 19], regularly cycling women, not on hormonal contraception, underwent two MRI scans: during their mid-follicular and late luteal menstrual phases. We investigated resting-state functional connectivity (Rs-FC), cortical thickness, and subcortical volumes of brain regions associated with the pathophysiology of BD and PMDD between groups, in the mid-follicular and late luteal phases of the menstrual cycle. All BD subjects were euthymic for at least 2 months prior to study entry.

**Results:**

Women in the BDPMDD group displayed greater disruption in biological rhythms and more subthreshold depressive and anxious symptoms through the menstrual cycle compared to other groups. Rs-FC was increased between the L-hippocampus and R-frontal cortex and decreased between the R-hippocampus and R-premotor cortex in BDPMDD vs. BD (FDR-corrected, *p* < 0.05). Cortical thickness analysis revealed decreased cortical thickness of the L-pericalcarine, L-superior parietal, R-middle temporal, R-rostral middle frontal, and L-superior frontal, as well as increased cortical thickness of the L-superior temporal gyri in BDPMDD compared to BD. We also found increased left-caudate volume in BDPMDD vs. BD (*p*_CORR_ < 0.05).

**Conclusion:**

Women with BD and comorbid PMDD display a distinct clinical and neurobiological phenotype of BD, which suggests differential sensitivity to endogenous hormones.

## Introduction

Bipolar Disorder (BD) affects approximately 1–4% of the population (1). It carries a significant burden of illness due to its chronicity, early age of onset, and severity (1, 2). Clinically, research shows that female reproductive events (e.g., postpartum period and menopause) have the potential to act as critical windows of mood worsening in women with BD (3–5). For instance, mood instability during the premenstrual phase in women with BD has been reported across numerous studies (6). Women with BD display high rates of premenstrual syndrome (PMS); with studies estimating that approximately 51–68% of women with BD report mood symptoms during the premenstrual period (7–10). Choi et al. investigated premenstrual exacerbation among women with BD to find that 51.6% of women with BD type-II displayed moderate to severe PMS as compared to 23.3% of women with BD type-I and 19.7% of healthy controls (9). Further, Fornaro and Perugi investigated the impact of premenstrual dysphoric disorder (PMDD) in a sample of 92 women with BD (11). In their sample, 27.2% of women met criteria for PMDD according to a semi-structured clinical interview. This subset of women with BD and comorbid PMDD displayed a higher number of Axis-I comorbidities than those without PMDD (11). Dias and colleagues conducted a large prospective study, which found that women with a diagnosis of BD and history of premenstrual exacerbation of mood displayed shorter time to relapse and greater symptom severity during 12 months of follow-up (12). Further, studies with a primary objective of examining prevalence of PMDD in community-based samples have also highlighted its association with BD (13). Wittchen and colleagues reported that women with a diagnosis of PMDD were eight times more likely to have a diagnosis of comorbid BD (13). It is important to note that smaller, underpowered studies have failed to find an association with BD and PMS (14–17). In a recent study of a large sample of women with BD (*N* = 1,099), our group found that women with comorbid PMDD had an earlier onset of bipolar illness, higher rates of rapid cycling, increased number of mood episodes, and higher rates psychiatric comorbidities (18). Notably, women with comorbid BD and PMDD had a shorter gap between BD onset and menarche, which points toward a potential link between puberty/sex hormones in the onset of BD in this population (18). Further, sex hormones have been implicated in the establishment and regulation of the HPA axis (19); literature supports dysregulation of this axis in BD (20), therefore suggesting that sex hormones may play a role in mediated an already dysregulated HPA axis in the case of BD.

Structural and functional MRI techniques are useful tools to investigate the neurobiological underpinnings of BD and potential vulnerabilities induced by the premenstrual period. Neurobiological models of BD propose that its pathophysiology is associated with dysregulation in neural pathways involved in emotional control and processing (21–24). Proposed models suggest a loss of top-down prefrontal modulation of limbic circuitry and aberrant functioning of two interrelated networks responsible for mediating emotional regulation: (1) lateral prefrontal-sub-cortical system [ventrolateral PFC, mid and dorsal anterior cingulate cortex (ACC), ventromedial striatum, globus paladus, and thalamus]; and (2) medial prefrontal-sub-cortical system (ventromedial PFC, subgenual ACC, nucleus accumbens, globus palladus, and thalamus) (22–24). Both of these networks function in synchrony to modulate the activity of the amygdala (23, 24). It has been hypothesized that an imbalance between these two neural streams may lead to the onset of affective episodes and emergence of clinical symptoms of BD.

Interestingly, there is considerable overlap between regions implicated in the neurobiology of BD, those affected by PMDD (25–27) and by female sex hormones (28, 29). Female sex hormones such as 17β-estradiol (E2) and progesterone (P4) bind to their specific receptors in various regions of the cerebral cortex and subcortical gray matter regions involved in emotional regulation and stress response, such as the amygdala, thalamus, hypothalamus, and hippocampal formation (30–32). Our group recently published a study investigating the hormonal correlates of resting-state functional connectivity (Rs-FC) across the menstrual cycle in healthy women. We found that serum levels of E2, P4, allopregnanlone, and dehydroepiandrosterone sulfate (DHEAS) correlated with patterns of resting-state functional coupling throughout the cerebral cortex (33). This contributes to a growing body of literature showing structural and functional brain changes across the menstrual cycle in women with and without PMDD [for review, see Ref. (27)]. Overall literature on women with PMDD is limited (27), but available studies using fMRI have found increased dlPFC activation during a working memory task, which also correlated with disease dimensions (26). Another study found increased fALFF in the bilateral precuneus, left hippocampus and inferior temporal cortex and decreased fALFF in the ACC and cerebellum in the late luteal phase of individuals with PMS (34). Overall, neurobiological models of PMDD suggest that there might be an increase in negative and decrease in positive emotional processing, and reduced top-down prefrontal modulation of limbic circuitry in the late luteal phase of the menstrual cycle (25, 35).

Despite emerging clinical literature suggesting a link between BD and PMDD, neuroimaging literature in these populations is sparse. To our knowledge, no brain imaging has been conducted in women with BD and comorbid PMDD. The primary goal of this study was to examine the brain structure and function in a group of well-characterized women with comorbid BD and PMDD using Rs-FC, cortical thickness analysis, and anatomical subcortical segmentation. We hypothesized that women with BD and comorbid PMDD would display menstrual cycle-related structural and functional differences in brain regions associated with emotional regulation compared to BD women without PMDD.

## Materials and Methods

### Participants

This study was carried out in accordance with the approval and recommendations of the Hamilton Integrated Research Ethics Board with written informed consent from all subjects. All subjects gave written informed consent in accordance with the Declaration of Helsinki. Participants were recruited through community advertisements in the Hamilton and Halton Regions, ON, Canada.

Eighty-five women between 16 and 45 years of age, with regular menstrual cycles (25–32 days) were enrolled. Participants were split into four groups based on their history of BD and PMDD: (1) healthy controls with no history of PMDD (CTRL); (2) women with PMDD but no other psychiatric diagnosis (PMDD); (3) BD with no history of PMDD (BD); and (4) BD with comorbid PMDD (BDPMDD).

Exclusion criteria included: (i) current or recent (previous 3 months) use of systemic hormonal treatment; (ii) pregnancy; (iii) contraindications for MRI; (iv) history of head trauma with loss of consciousness; (v) neurological disorders; (vi) current or recent (previous 6 months) history of alcohol or drug abuse or dependence; (vii) unstable general medical conditions. Regularly cycling women using levonorgestrel intrauterine device were allowed in the study due to its primarily localized hormonal effect. All women performed at least 2 months of prospective symptom charting using the Daily Record of Severity of Problems (DRSP) in order to confirm PMDD diagnosis, as per DSM-5 criteria (36). Study participants were informed that recreational drug use was not allowed during the course of study participation and all participants agreed to comply with this request. We did not perform drug testing on the day of the MRI scans.

Inclusion criteria for participants with BD (both BD and BDPMDD groups) included: (i) a diagnosis of BD according to the SCID-I (SCID-IV-TR) (37); (ii) no current depressive, manic, or hypomanic episodes according to the SCID-I; (iii) no changes in psychotropic medications or mood state within at least 2 months prior to and during the study. Due to the exceptionally high rates of comorbid psychiatric conditions in BD, lifetime but not current history of psychiatric comorbidities was allowed to provide a true reflection of most individuals with BD. Similarly, due to the high rates of comorbid psychiatric conditions in PMDD, a lifetime history of a single major depressive episode, past history of generalized anxiety disorder, or posttraumatic disorder was allowed so long as the individual was fully remitted for a minimum of 6 months prior to study entry.

Exclusion criteria for participants in the CTRL group included: (i) a lifetime history of any psychiatric disorder according to the SCID-I and (ii) greater than a 30% change in the four core symptoms of PMDD in their late luteal phase from their mid-follicular phase according to the DRSP (36). Women with BD without PMDD (BD group) also did not display greater than a 30% change in the four core symptoms of PMDD in their late luteal phase from their mid-follicular phase according to the DRSP. A diagnosis of PMDD (both in PMDD and BDPMDD groups) was established by two independent licensed psychiatrists blinded by subjects’ group status (Benicio N. Frey, Luciano Minuzzi) using 2 months of prospective daily symptom charting as confirmed by the DRSP (36).

### Study Design

Study participation was comprised of three visits to St. Joseph’s Healthcare Hamilton. The first visit consisted of administration of the SCID-I by a psychiatrist or trained Ph.D. candidate, followed by a psychiatric and gynecological history. The second and third visits took place during the mid-follicular phase (days 5–10 of the menstrual cycle) or the late luteal phase of the menstrual cycle (7 days preceding menses). Approximately half of the study participants began with their mid-follicular visit and half with their late luteal visit. Menstrual cycle phase was confirmed through prospective charting and hormonal analysis. Visits two and three included an MRI scan, collection of a blood sample for hormone analysis, and completion of validated clinical questionnaires as detailed below.

### Clinical Questionnaires

The Mongomery-Asberg Depression Rating Scale (MADRS) (38) and Hamilton Depression Rating Scale (HAMD) (39) were administered to assess severity of depressive symptoms. The Young Mania Rating Scale (YMRS) (40) was administered to assess the severity of manic/hypomanic symptoms through the menstrual cycle. Self-reported disruptions in biological rhythms were investigated using the Biological Rhythms Interview of Assessment in Neuropsychiatry (BRIAN) (41). The Pittsburgh Sleep Quality Index (PSQI) was used to asses sleep quality (42) and the State Trait Anxiety Inventory (STAI) was used to identify state and trait-based anxiety (43).

Statistical analysis of demographic and clinical variables was completed using R (Version 3.1.2^1^). Shapiro–Wilks tests and Bartlett’s test were used to evaluate normal distribution of clinical scales and homogeneity of variances between groups, respectively. Differences in demographic variables and clinical scales were analyzed using Kruskal–Wallis or Analysis of Variance (ANOVA). Pairwise between-group differences were ascertained using parametric and nonparametric Dunnett Tests using BDPMDD as the reference group. A *p*-value of < 0.05 was considered significant.

### Hormonal Analysis

Immediately following both MRI scans, 10 ml of whole blood was collected in serum tubes to confirm menstrual phase. The blood was clotted at room temperature for 45 min, and centrifuged at 20°C for 15 min at 3,000 rpm. Four serum aliquots were obtained and frozen at −80°C until assayed. Serum was assayed for progesterone (P4), 17-β-estradiol (E2), and DHEAS using commercially prepared solid phase enzyme-linked immunosorbent assay (ELISA) kits from ALPCO Diagnostics, Salem, NH, USA. Samples were also assayed for allopregnanolone (ALLO) using ELISA kit from Kiamiya Biomedical Company, Seattle, WA, USA. All serum samples were assayed in duplicate following the manufacturer’s protocol, with a fresh aliquot for each analyte. The inter-assay variations for P4, E2, DHEAS, and ALLO were 11.3, 8.7, 9.2, and 6.0%, respectively. The intra-assay variations were 10.4, 7.7, 9.3, and 11.7% and the sensitivities were 0.1 ng/ml, 10 pg/ml, 0.005 µg/ml, and 0.52 ng/ml, respectively. A licensed gynecologist (Dustin Costescu) confirmed that hormone levels were within physiological range for each menstrual phase.

### MRI Protocol

#### Image Acquisition

Images were acquired using a GE whole body short-bore 3T scanner with eight parallel receiver channels (General Electric, Milwaukee, WI, USA). Anatomical images were acquired with high-resolution T1-weighted images (gradient-echo inversion-recovery sequence, TR = 1.6 s, TE = 5 ms, matrix 256 × 256 × 128, FOV 220 mm × 220 mm, slice thickness 1 mm). Functional resting-state imaging was completed using a T2*-interleaved echo-planar imaging sequence with TR = 2,000 ms, TE = 40 ms, flip angle = 60°, 4 mm thick, 29 axial slices, matrix 64 × 64 resolution over 256 mm FOV. Once positioned in the scanner, participants were instructed to “Lay still, relax, and try not to think about anything in particular” as they looked at a fixation point. Anatomical and resting-state scans took place over 10 min and were followed by functional tasks that will be published at a later date.

#### Preprocessing for Rs-FC

The resting-state and anatomical MRI data were preprocessed using the Statistical Parametric Mapping Software SPM12^2^ and CONN Functional Connectivity Toolbox Verson 17e^3^ (44). Imaging data were obtained in DICOM file format and converted to NIFTI using SPM and then uploaded to CONN for further preprocessing. The default preprocessing pipleline for volume-based analyses was optimized to preprocess both structural and functional scans. Briefly, structural scans were translated and centered to (0, 0, 0) coordinates and subsequently underwent direct segmentation (gray matter, white matter, and cerebrospinal fluid) and Montreal Neurological Institute (MNI) normalization. Functional scans were realigned and unwarped (motion estimation and correction), and translated and centered to (0, 0, 0) coordinates. Outliers were identified as volumes with greater than 2 mm of motion in the translational plane, and were detected using the ART toolbox and added as condition files for denoising. Images with motion greater than 3 mm in the translational plane and 3° in the rotational plane were discarded. Finally, functional data were segmented (GM, WM, CSF), normalized to MNI space, and spatially smoothed to increase the signal to noise ratio with an 8-mm FWHM Gaussian filter.

#### Seed-Based Analysis

Seed-based analysis was completed using the CONN toolbox version 17e (44). Subject specific maps of CSF and WM were used as nuisance regressors during the denoising step of analysis. The aCompCor strategy was employed within CONN to control for the effects of physiological motion and residual head movement. The functional images were then temporally band-pass filtered (0.008–0.09 Hz). The following regions were used as seed points in a seed-to-voxel analysis: (i) right and left vlPFC; (ii) right and left vmPFC; (iii) right and left amygdala; (iv) right and left hippocampus; (v) right and left postcentral gyrus; (vi) precuneus; and (vii) subcallosal cortex. They were taken from the FSL Harvard-Oxford Atlas available with CONN. Statistical analysis was performed using an ANOVA in the mid-follicular and late luteal menstrual phases. Seed points with significant clusters were then explored using *post hoc* testing. A voxel height threshold of *p* < 0.001 and cluster threshold of 0.05-FDR corrected was used to identify significant clusters that responded to regions of interest. Due to previous studies showing an association between BMI and brain volume and chemistry in BD (45–48), and since BMI differed between groups, we tested any potential effects of BMI on Rs-FC by (i) adding BMI as a second-level covariate and (ii) directly analyzing the effect of BMI for particular regions of interest within groups. The same sub-analysis was completed for psychosis history and medication load, as these variables may influence Rs-FC.

Fisher transformed functional connectivity values for each subject were extracted from each significant cluster. Values were then exported to R (Version 3.1.2.) and correlated with luteal MADRS and STAI scores (state and trait) using spearman correlations.

#### Potential Effects of Medication on Rs-FC

Analyses were completed to assess the potential effects of psychotropic medications on Rs-FC in bipolar subjects according to previous imaging studies investigating dose equivalences (49–51). Antipsychotics, lithium, anticonvulsants, antidepressants, and anxiolytics were coded to 0, 1, or 2 to represent absent, low, or high dose medication groupings using the method described by Hassel and colleagues (49). Lithium, anticonvulsants, and antidepressants were categorized into groups from 1 to 4 depending on medication dose (51). Based on this, individuals in category 1 or 2 were grouped in the low dose group (scoring a 1), and individuals in category 3 or 4 were grouped in the high dose group (scoring a 2). Antipsychotics were converted to chlorpromazine equivalent doses. Doses below or above the mean effective daily dose of chlorpromazine were coded as 1 or 2, respectively. Individuals not taking antipsychotics were coded 0. Anxiolytics were also grouped into similar categories based on the recommended daily dosage found in the *Physician’s Desk Reference* (52). In this case, the dose was coded as 0 if absent or present in very low dose with reference to the midpoint, 1 if around the midpoint, and 2 if higher than the midpoint. Composite scores of medication load were then calculated and used as second-level covariates in CONN in an analysis using our *a priori* seed points.

#### Cortical Thickness and Subcortical Volume Analysis

Cortical thickness and subcortical volume analysis were conducted using the FreeSurfer Software Version 5.3.0.^4^ The FreeSurfer Software uses a semi-automated process, which is described in full detail in Ref. (53, 54). Briefly, non-brain tissue is removed through a process known as skull stripping, images are transformed into Talairach space, signal intensity is corrected and normalized, tissue is classified into WM, GM, and CSF, and the white matter and gray matter boundaries are tessellated. Each subject’s images were manually inspected to ensure that the GM/WM and GM/CSF boundaries were correctly identified. Images with inaccuracies in boundary identification were manually corrected and reprocessed. Then, data were normalized and smoothed using a 10-mm full-width-half-maximum Gaussian kernel. Cortical thickness was measured as the closest distance between the GM/WM boundary and the GM/CSF boundary at each vertex on the tessellated surface.

Statistical analysis was completed in FreeSurfer qdec. Cortical thickness was compared between groups using a general linear model using age and BMI as covariates, using a different slope different onset (DODS) approach. All clusters were required to have a minimum size of 50 mm^2^. All results were Bonferroni corrected for multiple comparisons.

The volumes of subcortical gray matter regions were extracted from FreeSurfer (automated segmentation) and corrected for intracranial volume. Volumes across regions of interest were compared between groups using an ANCOVA with BMI as a covariate and explored using *post hoc* analyses and multiple comparison corrections. Regions of interest were (i) left and right caudate; (ii) left and right putamen; (iii) left and right hippocampus; (iv) left and right amygdala; (v) left and right thalamus; (vi) left and right accumbens area; and (vii) left and right ventral diencephalon.

## Results

### Clinical Scales

Participants in the BDPMDD group had a significantly higher BMI than CTRL (*p* = 0.001) and PMDD (*p* = 0.009) groups. As a result, BMI was added as a covariate in all imaging analyses. Groups were matched by age (*p* = 0.054) and years of education (*p* = 0.09). Neither medication load (*p* = 0.86), age of onset (*p* = 0.81), or number of comorbid conditions (*p* = 0.94) were different between the BD and the BDPMDD groups (Table 1).

**Table 1:** Participants demographics.

	HC (*n* = 25)	PMDD (*n* = 20)	BD (*n* = 21)	BDPMDD (*n* = 19)	*p*-Value
Age (SD)	27.44 (7.74)	31.80 (7.33)	33.57 (8.04)	31.74 (7.91)	*p* = 0.054
BMI (SD)	23.24 (3.29)	24.02 (4.32)	26.76 (5.96)	29.46 (5.11)	*p* < 0.001
Years of education (SD)	16.94 (2.64)	17.23 (3.55)	16.40 (2.82)	15.05 (2.07)	*p* = 0.091
**Illness history**					
Age of onset (SD)			18.3 (7.74)	17.1 (5.85)	*p* = 0.817
Average number of Comorbidities (SD)			1.27 (1.35)	1.55 (1.68)	*p* = 0.943
Diagnosis			BD-I: 12 BD-II: 9	BD-I: 7 BD-II: 12	
History of psychosis			3	7	
**Psychiatric medications**					
Lithium			3	1	
Anticonvulsants			7	8	
Antipsychotics			10	8	
Anxiolytics			6	10	
Antidepressants		2	5	4	
Sleep aids			2	3	
Mean # of psychotropic meds (SD)			2.05 (1.02)	2.50 (1.22)	*p* = 0.868

*BD, bipolar disorder; BD-I, bipolar disorder type-I; BD-II, bipolar disorder type-II; BMI, body mass index; PMDD, premenstrual dysphoric disorder; BDPMDD, BD with comorbid PMDD*.

Consistent with research suggesting that comorbidity with PMDD is associated with an increased illness burden for women with BD (12, 18), we found a stepwise progression of severity of symptoms from the CTRL group to the BDPMDD group (Table 2). Notably, even though participants in both the BD and BDPMDD groups were clinically euthymic for a minimum of 2 months prior to entering and during the study, the BDPMDD group had significant higher MADRS (*p* = 0.037) and BRIAN scores (*p* = 0.007) during the follicular phase of the menstrual cycle. Hormonal levels were all within normal range for each menstrual phase, and there no between-group differences except for higher ALLO levels in the PMDD group (Table 3).

**Table 2:** Clinical scores between groups.

Clinical scale (SD)	HC	PMDD	BD	BDPMDD	*p*-Value
MADRS—follicular phase	2.60 (2.99)	5.40 (5.40)	6.33 (5.47)	10.79 (5.50)	Overall: *p* < 0.001 BDPMDD-BD: *p* = 0.037 BDPMDD-PMDD: *p* = 0.017 BDPMDD-CTRL: *p* < 0.001

MADRS—luteal phase	2.16 (2.90)	14.68 (7.80)	9.24 (6.86)	14.79 (7.28)	Overall: *p* < 0.001 BDPMDD-BD: *p* = 0.051 BDPMDD-PMDD: *p* = 0.997 BDPMDD-CTRL: *p* < 0.001

HAMD—follicular phase	1.44 (1.69)	3.25 (3.37)	4.43 (3.71)	6.58 (4.23)	Overall: *p* < 0.001 BDPMDD-BD: *p* = 0.22 BDPMDD-PMDD: *p* = 0.029 BDPMDD-CTRL: *p* < 0.001

HAMD—luteal phase	1.20 (1.58)	9.37 (5.12)	5.95 (3.97)	9.16 (4.50)	Overall: *p* < 0.001 BDPMDD-BD: *p* = 0.067 BDPMDD-PMDD: *p* = 0.993 BDPMDD-CTRL: *p* < 0.001

YMRS—follicular phase	0.48 (1.00)	0.75 (1.12)	1.48 (1.29)	1.37 (1.67)	Overall: *p* = 0.106

YMRS—luteal phase	0.60 (1.15)	1.89 (1.56)	1.38 (1.43)	2.53 (1.65)	Overall: *p* < 0.001 BDPMDD-BD: *p* = 0.103 BDPMDD-PMDD: *p* = 0.308 BDPMDD-CTRL: *p* < 0.001

BRIAN—follicular phase	27.28 (6.36)	35.45 (11.2)	37.48 (9.26)	47.79 (8.99)	Overall: *p* < 0.001 BDPMDD-BD: *p* = 0.007 BDPMDD-PMDD: *p* = 0.005 BDPMDD-CTRL: *p* < 0.001

BRIAN—luteal phase	28.48 (8.41)	40.61 (12.3)	38.35 (12.4)	48.50 (8.42)	Overall: *p* < 0.001 BDPMDD-BD: *p* = 0.009 BDPMDD-PMDD: *p* = 0.109 BDPMDD-CTRL: *p* < 0.001

PSQI—follicular phase	4.28 (2.42)	5.05 (2.87)	5.95 (3.03)	7.95 (3.61)	Overall: *p* = 0.003 BDPMDD-BD: *p* = 0.165 BDPMDD-PMDD: *p* = 0.032 BDPMDD-CTRL: *p* = 0.003

STAI-state—follicular phase	29.16 (6.84)	31.80 (9.47)	33.40 (9.49)	40.58 (14.2)	Overall: *p* = 0.028 BDPMDD-BD: *p* = 0.209 BDPMDD-PMDD: *p* = 0.160 BDPMDD-CTRL: *p* = 0.028

STAI-state—luteal phase	31.04 (7.27)	43.47 (13.1)	34.10 (9.19)	45.84 (14.0)	Overall: *p* = 0.005 BDPMDD-BD: *p* = 0.024 BDPMDD-PMDD: *p* = 0.950 BDPMDD-CTRL: *p* = 0.005

STAI-trait—follicular phase	30.08 (7.28)	33.95 (9.19)	41.70 (10.2)	47.63 (15.2)	Overall: *p* = 0.002 BDPMDD-BD: *p* = 0.286 BDPMDD-PMDD: *p* = 0.014 BDPMDD-CTRL: *p* = 0.002

STAI-trait—luteal phase	30.67 (9.43)	38.63 (11.7)	41.95 (11.6)	48.63 (14.0)	Overall: *p* < 0.001 BDPMDD-BD: *p* = 0.28 BDPMDD-PMDD: *p* = 0.070 BDPMDD-CTRL: *p* < 0.001

*BRIAN, Biological Rhythms Interview of Assessment in Neuropsychiatry; HAMD, Hamilton Depression Rating Scale; MADRS, Montgomery Asberg Depression Rating Scale; PSQI, Pittsburgh Sleep Quality Index; STAI, State Trait Anxiety Inventory; YMRS, Young Mania Rating Scale; BD, bipolar disorder; PMDD, premenstrual dysphoric disorder; BDPMDD, BD with comorbid PMDD*.

**Table 3:** Hormone levels in the mid-follicular and late luteal menstrual phases.

Hormone levels	HC	PMDD	BD	BDPMDD	*p*-Value
**Follicular phase**					
P4 (pg/mL)	1.66 (2.08)	1.23 (0.91)	1.66 (4.15)	1.03 (0.84)	*p* = 0.127
E2 (ng/mL)	69.71 (45.2)	87.27 (38.2)	73.71 (57.3)	76.34 (51.6)	*p* = 0.220
ALLO (ng/mL)	4.13 (1.60)	13.58 (14.8)	4.97 (4.01)	5.82 (4.25)	*p* = 0.010
DHEAS (μg/dL)	163.56 (77.2)	161.67 (93.1)	160.63 121.9	129.27 (79.2)	*p* = 0.506

**Luteal phase**					
P4 (pg/mL)	4.53 (2.82)	3.72 (2.10)	5.25 (11.0)	4.20 (3.62)	*p* = 0.505
E2 (ng/mL)	86.54 (51.3)	106.13 (50.7)	87.18 (73.0)	78.77 (39.5)	*p* = 0.187
ALLO (ng/mL)	4.88 (2.02)	13.45 (11.7)	5.54 (4.16)	5.58 (4.78)	*p* = 0.012
DHEAS (μg/dL)	155.92 (73.9)	149.86 (75.9)	166.14 (156.8)	124.44 (84.3)	*p* = 0.605

*ALLO, allopregnanolone; E2, β17-estradiol; DHEAS, dehydroepiandrosterone sulfate; P4, progresterone; BD, bipolar disorder; PMDD, premenstrual dysphoric disorder; BDPMDD, BD with comorbid PMDD*.

### Resting-State fMRI

**Figure 1:**
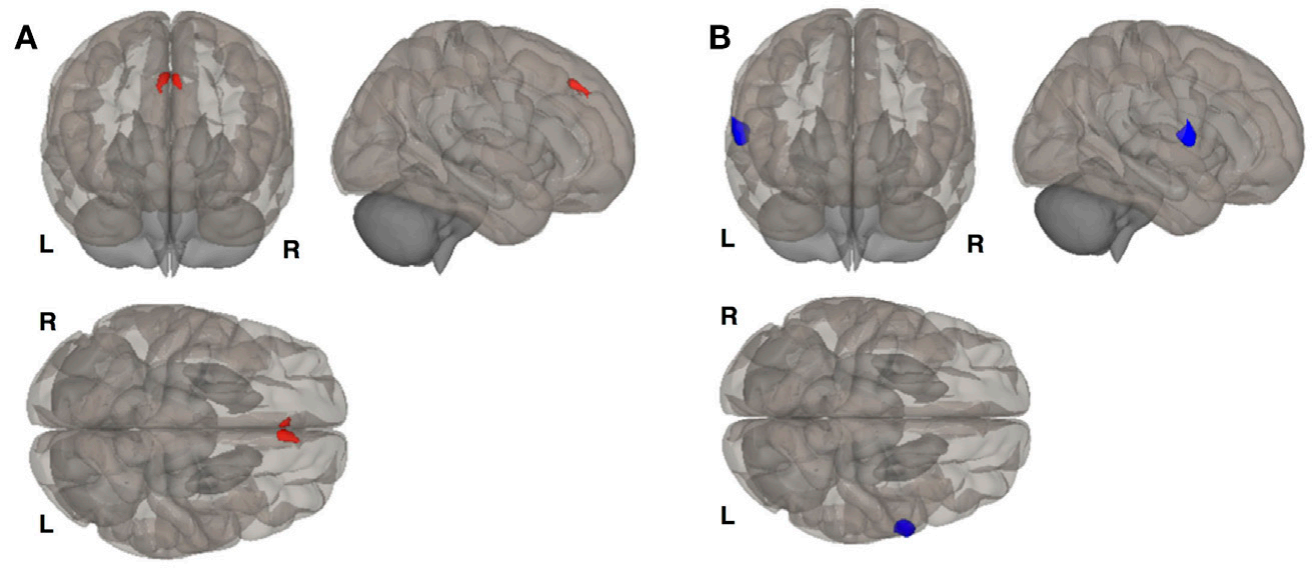
Clusters of resting state functional connectivity between bipolar disorder (BD) and BD with comorbid PMDD (BDPMDD) groups in the late luteal phase. **(A)** Increased connectivity in between the left hippocampus (seed) and right frontal cortex (*k* = 165; *X* = + 02, *Y* = + 34, *Z* = + 44; *p* = 0.048, FDR-corrected), BDPMDD compared to BD. **(B)** Decreased connectivity between the right hippocampus (seed) and the left premotor cortex (*k* = 201; *X* = + 64, *Y* = + 02, *Z* = + 06; *p* = 0.029, FDR-corrected) in BDPMDD compared to BD. Statistical details are shown in Table 4.

We investigated whole brain differences in Rs-FC using a seed-to-voxel approach. We found significant group differences in Rs-FC particularly in the late luteal phase using the right and left hippocampus as seed points (Table 4, Figure 1). Specifically, we found differences in functional connectivity between the right hippocampus and the right premotor cortex (BA6) (*k* = 166; *x* = +64, *y* = +02, *z* = +06; *p* = 0.044, FDR-corrected). In addition, we found the greatest number of clusters when using the left hippocampus as a seed point, which yielded three significant clusters: (i) right somatosensory association cortex (BA5) (*p* = 0.037, FDR-corrected); (ii) right frontal cortex (BA8) (*p* = 0.037, FDR-corrected); (iii) right somatosensory cortex (BA1) (*p* = 0.033, FDR-corrected). Pairwise analyses revealed decreased connectivity between the right hippocampus and the left premotor cortex (*p* = 0.029, FDR-corrected) in BDPMDD compared to BD, and increased connectivity in BDPMDD compared to BD between the left hippocampus and the right frontal cortex (*p* = 0.048, FDR-corrected) in the late luteal phase (Figure 1).

**Table 4:** Differences in Rs-FC across groups.

Seed region	Group/change	Area	Coordinates (*x* *y* *z*)	*T* min	Cluster size	Clusterwise *p*-value
Luteal phase						
**R-Hippocampus**						
	BDPMDD > PMDD	L-Frontal Cortex (BA 8) R-Dorsolateral Prefrontal Cortex (BA 9) L-Dorsolateral Prefrontal Cortex (BA 9)	−42 +16 +44 +12 +42 +40 −20 +44 +42	3.33	539 276 197	*p* = 0.0004 *p* = 0.011 *p* = 0.031
	BDPMDD < PMDD	R-Primary Motor Cortex (BA 4)L-Primary Motor Cortex (BA 4)	+66 +00 +14 −50 −08 +12	−3.33	297 229	*p* = 0.007 *p* = 0.011
	BD > BDPMDD	R-Premotor Cortex (BA 6)	+64 +02 +06	3.32	201	*p* = 0.029

**L-Hippocampus**						
	BDPMDD > BD	R-Frontal Cortex (BA 8)	+02 +34 +44	3.32	165	*p* = 0.048
	BDPMDD < PMDD	L-Somatosensory Cortex (BA 1)	−60 −18 +50	−3.33	431	*p* = 0.016

*Peak cluster coordinates are shown in Montreal Neurological Institute coordinates. BA, Brodmann Area; L, Left; R, Right; BD, bipolar disorder; PMDD, premenstrual dysphoric disorder; BDPMDD, BD with comorbid PMDD*.

We found that MADRS scores were positively correlated with Rs-FC between the right hippocampus and left premotor cortex (*r* = 0.46, *p* = 0.037) in BD but not BDPMDD. Further, STAI-state scores were positively correlated with clusters between the left hippocampus and right dlPFC [BA9] (*r* = 0.54, *p* = 0.017) and right dorsal frontal cortex [BA8] (*r* = 0.46, *p* = 0.048) in BDPMDD but not PMDD.

Moreover, we found increased functional connectivity between the right hippocampus and clusters in the left frontal cortex, right dorsolateral prefrontal cortex, and left dorsolateral prefrontal cortex in BDPMDD compared to PMDD. Further, using the same seed point decreased functional connectivity was found with bilateral clusters in the right and left primary motor cortex in BDPMDD compared to PMDD. Decreased functional connectivity between the left hippocampus and a cluster in the left somatosensory cortex was also found in BDPMDD compared to PMDD (Table 4).

No differences in Rs-FC were found using the following seed points: (i) right and left vlPFC; (ii) right and left vmPFC; (iii) right and left amygdala; and (iv) right and left postcentral gyrus. We tested and we did not find any significant effect of medication load, psychosis, or BMI on any of the Rs-FC results.

### Cortical Thickness

**Figure 2:**
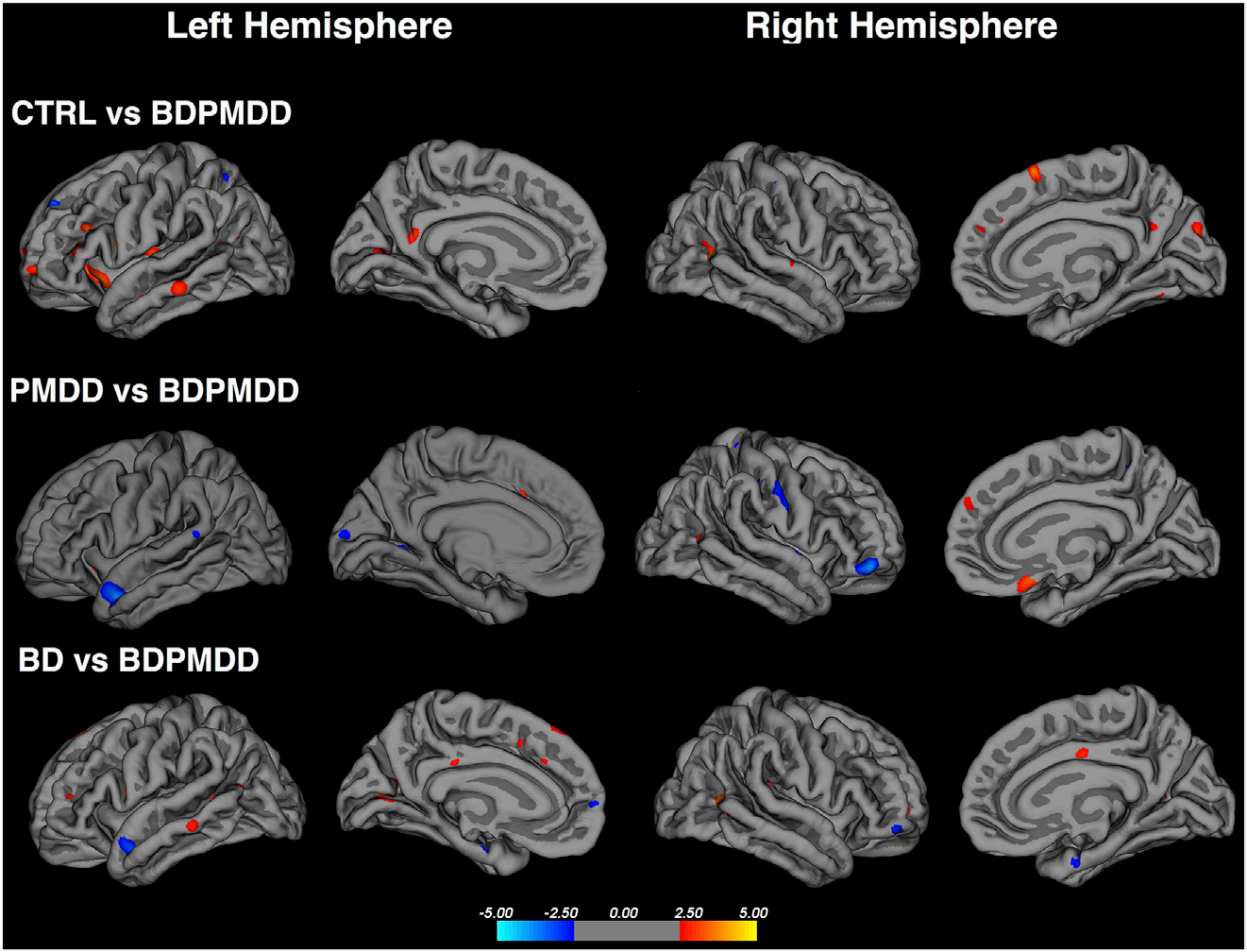
Cortical thickness across groups compared to BD with comorbid PMDD (BDPMDD). Regional differences in cortical thickness are shown with reference to the first group in the comparison. Blue regions represent decreased cortical thickness; Red regions represent increased cortical thickness. Further details are shown in Table 5.

We identified six clusters representing significant differences in cortical thickness between BD and BDPMDD groups (Table 5, Figure 2). Compared to BD, BDPMDD women displayed cortical thinning in the following regions: (i) left pericalcarine gyrus (*p*_CORR_ = 0.007); (ii) left superior parietal gyrus (*p*_CORR_ = 0.0024); (iii) right middle temporal gyrus (*p*_CORR_ = 0.0002); (iv) right rostral middle frontal gyrus (*p*_CORR_ = 0.0006); and (v) left superior frontal gyrus (*p*_CORR_ = 0.04). Further, we found increased thickness in BDPMDD compared to BD in the left superior temporal gyrus (*p*_CORR_ = 0.045).

**Table 5:** Differences in cortical thickness between groups.

	Size mm^2^	Peak coordinates *x* *y* *z*	Peak *T* value	*p*_CORR_Value
**CTRL vs. BDPMDD**				
CTRL > BDPMDD				
L-Insula	262.25	−32.1 14.7 −7.0	4.0548	0.0022
R-Middle temporal	220.89	45.6 −61.0 6.4	4.7045	0.0004
R-Medial orbitofrontal	148.09	11.1 28.0 −17.8	3.5026	0.0048
L-Pars triangularis	91.66	−46.0 32.6 8.4	3.3432	0.0209
L-Rostral middle frontal	85.62	−40.2 27.7 23.7	3.5982	0.0099
R-Cuneus	80.53	7.5 −83.8 24.4	2.6684	0.0444
R-Superior frontal	69.04	7.6 17.0 59.0	3.5556	0.004

**PMDD vs. BDPMDD**				
PMDD > BDPMDD				
R-Medial oribitofrontal	126.21	12.6 26.6 −16.2	3.5949	0.0060
R-Medial orbitofrontal	58.92	7.0 15.9 −15.4	3.0872	0.0246
R-Inferior parietal	56.52	44.0 −61.7 6.6	3.0320	0.0282
BDPMDD > PMDD				
L-Superior Temporal	215.50	−48.3 4.9 −24.7	3.5025	0.0039
R-Pars Orbitalis	137.85	43.8 44.6 −9.7	3.7023	0.0048
L-Lingual	100.15	−23.5 −59.9 0.1	3.3728	0.0057
R-Superior parietal	86.37	26.7 −69.2 28.6	2.9995	0.0306

**BD vs. BDPMDD**				
BD > BDPMDD				
L-Pericalcarine	417.43	−13.1 −74.0 2.8	3.5462	0.0072
L-Superior parietal	236.87	−20.7 −62.4 36.5	3.9530	0.0024
R-Middle temporal	223.90	45.6 −61.0 6.4	4.8981	0.0002
R-Rostral middle frontal	105.38	25.3 49.3 0.3	4.0143	0.0006
L-Superior frontal	88.44	−10.0 34.2 50.9	2.8887	0.0402
BDPMDD > BD				
L-Superior temporal	112.82	−48.7 4.8 −24.0	2.8448	0.045

*L, left; R, right; PMDD, premenstrual dysphoric disorder; BD, bipolar disorder; BDPMDD, BD with comorbid PMDD*.

We found seven regions of cortical thinning in BDPMDD compared to controls: (i) left insula; (ii) right middle temporal gyrus; (iii) right medial orbitofrontal gyrus; (iv) left pars triangularis; (v) left rostral middle frontal gyrus; (vi) right cuneus; and (vii) right superior frontal gyrus (all *p*_CORR_ < 0.05). Compared to PMDD the BDPMDD group had increased cortical thickness in the (i) left superior temporal gyrus; (ii) right pars orbitalis; (iii) left lingual gyrus; and (iv) right superior parietal gyrus and decreased cortical thickness in the (i) right medial orbitofrontal gyrus and (ii) right inferior parietal gyrus (all *p*_CORR_ < 0.05) (Table 5, Figure 2). There were no within-group differences in cortical thickness across the mid-follicular and late luteal menstrual phases.

### Subcortical Volumes

We investigated the volume of subcortical regions of interest between groups in each menstrual phase, including right and left caudate; left and right putamen; left and right hippocampus; left and right amygdala; left and right thalamus; left and right accumbens area; and left and right ventral diencephalon. We found significant between-group differences in the left caudate during the late luteal menstrual phase. *Post hoc* analyses revealed that these results were driven by increased volume in the left caudate in BDPMDD compared to BD (*p*_CORR_ = 0.007). We did not find any other differences in the volume of other regions of interest in either menstrual phase between groups.

## Discussion

The main finding from our seed-to-voxel analysis was that women with BD and comorbid PMDD displayed distinct patterns of Rs-FC compared to those with BD without a diagnosis of PMDD, particularly in a network involving the left and right hippocampus and premotor and frontal cortical areas. Interestingly, these differences in Rs-FC were observed specifically during the (symptomatic) late luteal phase of the menstrual cycle. Structural analyses revealed differences in cortical thickness between BD and BDPMDD groups across parietal, temporal, and frontal regions, and a specific increased volume in the left caudate. These neuroimaging findings may highlight a biological susceptibility in women with comorbid BD and PMDD that predisposes or contributes to a worse course of their bipolar illness (18). In addition, given that estrogen receptor alpha and beta are highly expressed in the hippocampus and the temporal cortex (32, 55), these areas may be a target for future studies investigating whether the manipulation of hormonal levels (e.g., oral contraceptives) can modulate these networks and ameliorate the premenstrual worsening observed in women with comorbid BD and PMDD (5, 56).

### Clinical Scales

We found that the BDPMDD group reported significantly higher scores than all of the other groups in most of the clinical questionnaires. This is of particular importance as in our study all women with a diagnosis of BD were clinically euthymic for a minimum of 2 months before study entry. The presence of subthreshold depressive and anxious symptoms, as well as disturbances in sleep and biological rhythms during the mid-follicular phase may render these women more vulnerable to relapse. This is supported by literature that highlights that the severity of circadian rhythm disruptions and subthreshold symptoms are associated with higher risk of relapse (57–62). For instance, a systematic review by Jackson et al. found that disruptions in sleep were the most common prodrome of mania and sixth of bipolar depression (63). In addition, a greater severity of subthreshold symptoms has also been associated with poorer psychosocial functioning (58). This may highlight susceptibility of women with BD and comorbid PMDD to develop more frequent mood episodes and contribute to their burden of illness. These findings support a study by Dias et al., which found that women with BD and premenstrual mood worsening represent a phenotype of BD that is more symptomatic and relapse prone (12). This is also consistent with our recent study showing that women with BD and comorbid PMDD had a larger number psychiatric comorbidities, higher rates of rapid cycling, and increased number of hypo/manic and depressive episodes (18).

### Resting-State Functional Connectivity

We identified differences in Rs-FC between BD and BDPMDD groups using a seed-to-voxel analysis using the right and left hippocampus as seed points in the late luteal phase. Aberrant prefrontal-limbic circuitry is well documented in BD; many of the brain regions in this circuitry are also influenced by hormonal fluctuations and implicated in the pathophysiology of PMDD—the hippocampus as a primary example. The hippocampus is central to the trait-based pathology of BD and to the regulation of memory (encoding and retrieval) and emotional processes (21, 24, 64, 65). It also is a prominent site of E2 and 5HT receptors (31, 66), has high expression of BDNF (67) and plays an inhibitory role in stress response (68) and HPA axis regulation (20). Interestingly, it has been postulated that sex hormones interact with the HPA axis to facilitate the response to chronic stress (69). On the other hand, stress hormones may downregulate the action of sex hormones, by exerting modulatory effects on the reproductive neuroendocrine axis (19). Abnormalities in the HPA axis are also present in patients with BD and may have important consequences on the neurobiology of BD (20). The activity of the HPA axis is modulated by the hippocampi, prefrontal, and orbitofrontal cortices, ACC and amygdala—all of which have been implicated in the pathophysiology of BD (20, 24). Thus, considering that the women enrolled in our study were clinically stable, it is conceivable that the differences functional connectivity that we observed in the right and left hippocampi of women with comorbid BD and PMDD may be either an attempt from the brain to regulate the network involved in stress response, or a trait abnormality that may render these women more vulnerable to develop future episodes. Longitudinal studies are required to test these hypotheses. Literature also suggest that progesterone-derived neurosteroids may play an important role in mediating neuronal plasticity neural survival and neurogenesis in the hippocampus (67). Future neuroimaging studies correlating sex hormones with brain structure and function in BD and PMDD would be useful.

Structural changes have been observed in the hippocampus across the menstrual cycle as studies in healthy women report both decreased gray matter in the anterior hippocampus and increased volume in the bilateral hippocampi during the follicular phase (70, 71). fMRI studies in healthy women highlight increased functional coupling at rest between the bilateral hippocampi and bilateral superior parietal lobe in the late vs. early follicular phase, and decreased activity of the hippocampus in the luteal phase with response to emotional faces (72). In this context, the increased functional coupling between the left hippocampus and right frontal cortex in BDPMDD compared to controls may suggest (i) compensatory changes in functional connectivity resulting from the late luteal menstrual phase in women during the “hormonal sensitivity” period (premenstrual) and/or (ii) a functional trait marker of the added weight of PMDD on the pathophysiology of BD.

Interestingly, we found decreased coupling between the right hippocampus and the left premotor cortex in the late luteal phase in BDPMDD compared to BD. Previous studies using PET imaging have reported increased activity of the premotor cortex during “sadness” inducing paradigms in remitted but not depressed patients with BD (73), but decreased activity in patients with major depressive disorder (74). Further, a recent study reported that decreased connectivity of the somatomotor network, including the premotor cortex (as seen in the BDPMDD group), is present in bipolar depression and may represent aberrantly slow flow of inner time—a common feature of depression (75). Therefore, we hypothesize that this pattern of functional decoupling in BD and PMDD vs. BDPMDD may highlight the impact of comorbid PMDD in the late luteal phase in women with BD. Together, these findings suggest that, premenstrually, women with BD and comorbid PMDD may display changes in Rs-FC that mirror the ones observed in bipolar depression.

Compared to PMDD, increased functional connectivity between the right hippocampus and left frontal and bilateral dlPFC, and decreased functional connectivity between the right hippocampus and bilateral primary motor cortex, and left hippocampus and left somatosensory cortex in BDPMDD, further highlights involvement of somatomotor and executive control networks in the pathology of BD. These patterns of functional connectivity may reflect activity that is abnormally enhanced between limbic and frontal regions, and impaired between limbic and somatosensory and motor regions which play a role in mediating affective regulation and risk-taking behavior, both of which are affected in BD (76). Thus, aberrant patterns of functional connectivity between BDPMDD and PMDD in the late luteal phase may reflect functional differences related to the pathology of BD, and/or the pathology of comorbid PMDD and BD on brain functioning compared to PMDD. These findings also emphasize the need for more research exploring the impact of the endogenous hormones on women with BD.

### Structural MRI: Cortical Thickness and Automated Subcortical Segmentation

We found decreased thickness in several frontal and temporal gyri central to the default mode and cognitive networks in BDPMDD compared to CTRL, PMDD and BD. The only exceptions were the left superior temporal gyrus, which was thicker in individuals with BDPMDD compared to BD and PMDD, and the right pars orbitalis, left lingual, right superior parietal gyri compared to the PMDD group. A systematic review of cortical thickness studies in BD reported decreases in thickness in the bilateral superior frontal gyri, superior parietal gyrus, middle temporal gyrus and pericalcarine gyri compared to controls (77). As these regions are thinner in individuals in the BDPMDD group than BD and CTRL groups, this suggests, again, that comorbid PMDD may impose a worse impact on brain structure in women with BD. This also may help explain the affective lability reported in our clinical data and in literature in women with BD and comorbid PMDD. This was further reinforced as women in the BDPMDD group had increased gray matter volume of the caudate nucleus compared to the BD group. The caudate nucleus facilitates cross talk between prefrontal–cortical networks and subserves behavioral adaptations required for achievement of complex goals (23, 78). Studies investigating caudate volume between BD and controls have reported mixed findings with no differences (79, 80) and decreased volume compared to controls (81). Decreased volume of the caudate has also been reported in women with major depressive disorder (82), this may suggest that women with comorbid BD and PMDD may display structural and functional brain changes that mirror aspects of depression. Moreover, our seed-to-voxel analysis results show increased prefrontal-limbic activity, which neurobiological models of BD postulate may be mediated by both the dorsal and ventral prefrontal–cortical networks involving the striatum. When taken in conjunction with our Rs-FC findings, increased caudate volume in BDPMDD could also potentially reflect abnormal activity of prefrontal–cortical circuitry central to the trait-based pathology of BD, which seems to be exacerbated in the late luteal phase.

### Limitations

The limitations of our study deserve attention. First, the DRSP is a self-administered tool used to chart symptoms of PMS across the menstrual cycle (36). It is possible that women may provide an inaccurate account of their premenstrual symptoms or that use of the DRSP may be confounded by stressful life events. In both groups with a diagnosis of BD (BD and BDPMDD groups), symptoms reported on the DRSP may also be confounded by exacerbation of their bipolar illness. However, the use of mood, sleep, and biological rhythms questionnaires in both menstrual phases increased our confidence in self-reported results.

Second, Rs-fMRI provides an indirect measure of spontaneous neuronal activity in the ultralow frequency range (0.01–0.10 Hz) (83). The fMRI techniques used in this paper, seed-to-voxel and ROI-ROI are based on an oversimplification that BOLD activation measured is static through the duration of the scanning paradigm (84). Further, results may be confounded by the participant’s ability to remain awake for the duration of the scan, and inability to control the participant’s memory in the scanner. Although we advised participants not to think about anything in particular, and remain awake with their eyes fixed at the fixation point throughout the entire resting-state brain scan, no objective measures, such as simultaneous electroencephalogram or eye tracking, were in place to confirm that participants followed our instructions. Third, the effects of psychotropic medications on Rs-FC are a common limitation of fMRI research in BD (85). Studies investigating Rs-FC in BD, including our current and previous work (86), have largely ruled out the influence of medication following correlation analysis with BOLD activation (87–89). It would be almost impossible to recruit a sizeable sample of BD subjects in sustained clinical remission without treatment. The primary goal of this study was to investigate brain structure and function of comorbid BD and PMDD. We believe this goal could have only been achieved with a primarily medicated sample, by ensuring that all participants with BD were euthymic throughout the study (37). Another limitation of our sample was that it comprised both women with BD type-I and BD type-II. We encourage future work on differences in Rs-FC between women with BD type-I and BD type-II. Finally, it is important to note that our study was cross-sectional and as a result this study only reflects a current picture of disease state and not disease progression. Longitudinal studies in this area are required to provide a picture of disease burden and progression in women with BD and comorbid PMDD.

In conclusion, we found differences in the thickness of the cerebral cortex in regions critical to emotional regulation and cognition, as well as volumetric enlargement of the left caudate in the late luteal phase of participants in the BDPMDD group compared to BD group. Further, results from Rs-FC analysis highlight differences in brain regions dense in E2 and 5HT receptors, such as the right and left hippocampus and the temporal cortex, which may be liable to hormonal influence. Such differences in brain structure and function may help explaining the worse course of illness and clinical profile in women with these comorbid conditions. When taken in the context of other literature on this population, our results suggest that women with BD and comorbid PMDD display a distinct clinical and neurobiological phenotype of BD involving sensitivity to endogenous hormones.

## Ethics Statement

This study was carried out in accordance with the recommendations of the Hamilton Integrated Research Ethics Board with written informed consent from all subjects. All subjects gave written informed consent in accordance with the Declaration of Helsinki.

## Author Contributions

SS completed data collection, data analysis (clinical and neuroimaging), and wrote the manuscript. OA aided with participant recruitment. DC contributed to analysis of hormone data. DC, GH, LM, and BF contributed to text composition. LM and BF contributed to the study design and all aspects of analysis. MS, BF, and LM aided with scoring of the Daily Record of Severity of Problems. All authors approved the final version of the manuscript.

## Conflict of Interest Statement

The authors declare that the research was conducted in the absence of any commercial or financial relationships that could be construed as a potential conflict of interest.

## Footnotes

^1^https://www.r-project.org.

^2^http://www.fil.ion.ucl.ac.uk/spm.

^3^https://www.nitrc.org/projects/conn.

^4^http://surfer.nmr.mgh.harvard.edu/.
